# C-856-03. Implementation of Universal Screening for Bullying in a Pediatric Burn Clinic

**DOI:** 10.1093/jbcr/irag033.095

**Published:** 2026-04-06

**Authors:** Chinaemelum C Akpunonu, Samantha A Kesselring, Andrea A Wojtowicz, Hannah Kerby, Renata Fabia, Rajan K Thakkar, Kara Paulk, Babetta B Mathai, Dana M Schwartz

**Affiliations:** The Ohio State University Wexner Medical Center, Columbus, OH; Nationwide Children's Hospital, Columbus, OH; Nationwide Children's Hospital, Columbus, OH; Nationwide Children's Hospital, Columbus, OH; Nationwide Children's Hospital, Columbus, OH; Nationwide Children's Hospital, Columbus, OH; Nationwide Children's Hospital, Columbus, OH; Nationwide Children's Hospital, Columbus, OH; Nationwide Children's Hospital, Columbus, OH

## Abstract

**Introduction:**

Bullying in the United States has become an increasingly pervasive issue, with about 19.2% of children aged 12-18 years-old reporting experiences of bullying. Our ABA-Verified Pediatric Burn Center screens all patients for post-traumatic stress. Burn patients may be at increased risk for experiencing bullying, due to the cosmetic effects of the injury, making this an important population in whom to standardize bullying screening. Additionally, psychologists are integrated into our burn clinic and are well-positioned to address positive bullying screens with patients and families. The aim of our quality improvement project was to increase screening from 0 to 75% by the end of 2025, and sustain for 6 months, while providing resources and further interventions, if indicated.

**Methods:**

Using Institute for Healthcare Improvement Model for Improvement QI methodology, including developing a key-driver diagram, process map, and Plan-Do-Study-Act cycles, we developed a sustainable, universal bullying screening process for outpatient burn clinic. We created a 5-question bullying screen that was administered to all clinic patients, ages 5-19 years-old, and repeated at 3-month intervals for patients following up long-term. Psychology providers delivered standardized bullying interventions, in addition to appropriate patient-specific care, during patients’ burn clinic visits. Patient demographics and screening results were entered in a burn outpatient registry, which was queried from July 2024 to May 2025.

**Results:**

During this period, 105 unique patients (ages 5-19 years old) had clinic visits eligible for bullying screening. A total of 53.3% of patients were female, with a median age of 10.8 years (IQR 7.9-13.7). Overall, 70.5% of children (n = 74) were screened for bullying, with 20.3% (n = 15) reporting having experienced bullying. Within the group that screened positive, the median age was 10.6 years (IQR 8.3-12.2), 60% were White, and 60% were female. Psychology met with 93% (n = 14) of patients with positive screen to further delineate their bullying experiences and offer resources such as printed resources and mental health provider contacts.

**Conclusions:**

Implementation of universal screening for bullying in a pediatric burn clinic is feasible and effective. Having an embedded psychologist made this clinic particularly appropriate for screening, as we were poised to intervene on positive screens in a timely fashion. Patients who experience a burn injury, with effects on long-term appearance and function, are likely vulnerable to bullying. Next steps include measuring bullying experience at sequential visits to determine if burn injury affects bullying rate.

**Applicability of Research to Practice:**

A pediatric burn clinic, with high patient volume and embedded psychology resources, is an ideal setting to screen for bullying experience and deliver effective intervention.

**Funding for the Study:**

N/A.

